# Functioning of a Shallow-Water Sediment System during Experimental Warming and Nutrient Enrichment

**DOI:** 10.1371/journal.pone.0051503

**Published:** 2012-12-11

**Authors:** Christian Alsterberg, Kristina Sundbäck, Stefan Hulth

**Affiliations:** 1 Department of Biology and Environmental Sciences, University of Gothenburg, Göteborg, Sweden; 2 Department of Chemistry and molecular biology, University of Gothenburg, Göteborg, Sweden; University of Southampton, United Kingdom

## Abstract

Effects of warming and nutrient enrichment on intact unvegetated shallow-water sediment were investigated for 5 weeks in the autumn under simulated natural field conditions, with a main focus on trophic state and benthic nitrogen cycling. In a flow-through system, sediment was exposed to either seawater at ambient temperature or seawater heated 4°C above ambient, with either natural or nutrient enriched water. Sediment–water fluxes of oxygen and inorganic nutrients, nitrogen mineralization, and denitrification were measured. Warming resulted in an earlier shift to net heterotrophy due to increased community respiration; primary production was not affected by temperature but (slightly) by nutrient enrichment. The heterotrophic state was, however, not further strengthened by warming, but was rather weakened, probably because increased mineralization induced a shortage of labile organic matter. Climate-related warming of seawater during autumn could therefore, in contrast to previous predictions, induce shorter but more intensive heterotrophic periods in shallow-water sediments, followed by longer autotrophic periods. Increased nitrogen mineralization and subsequent effluxes of ammonium during warming suggested a preferential response of organisms driving nitrogen mineralization when compared to sinks of ammonium such as nitrification and algal assimilation. Warming and nutrient enrichment resulted in non-additive effects on nitrogen mineralization and denitrification (synergism), as well as on benthic fluxes of phosphate (antagonism). The mode of interaction appears to be related to the trophic level of the organisms that are the main drivers of the affected processes. Despite the weak response of benthic microalgae to both warming and nutrient enrichment, the assimilation of nitrogen by microalgae was similar in magnitude to rates of nitrogen mineralization. This implies a sustained filter function and retention capacity of nutrients by the sediment.

## Introduction

With an ongoing environmental change, the structure and function of aquatic ecosystems are challenged, not only by local anthropogenic stressors such as eutrophication, toxicants and physical disturbance, but also by global stressors such as increased seawater temperature and acidification [1], [2]. Studies investigating stressor-related responses on organisms or ecosystems often include one individual stressor, even though additional stressors can be expected to affect the response of the first stressor, resulting in non-additive interactive effects [2], [3]. For example, it has been suggested that future warming will alter food-web structure and ecosystem functioning [4], [5] and exacerbate the effect of other stressors, such as ocean acidification and toxicants [6], [7]. Even though there are a large number of studies on multiple stressors on species level [2], the number of studies under ecologically more relevant conditions has started to increase only during the last decade [8], [9], [10]. Thus, it is still difficult to draw any general conclusions about the importance of various types of interactions (synergistic, antagonistic or additive) in coastal marine ecosystems [2]. This is particularly true for shallow-water sediment systems [2], [11].

At the interface between land and sea, illuminated shallow-water sediment systems significantly contribute to the total primary production of coastal marine ecosystems and function as coastal nutrient filters [12], [13]. In these areas, processes that are crucial for ecosystem functioning (primary production, mineralization of organic material and nutrient cycling) mainly occur in the sediment and largely depend on microscopic organisms, such as microalgae, bacteria and archaea [12]. The biofilm of benthic microalgae constitutes an important functional part of the coastal filter by transforming, storing, and removing nutrients from the overlying water [13]. Previous experiments in seagrass and planktonic environments have shown interactive, mainly additive effects from simultaneous warming and nutrient enrichment, such as massive blooms of macro- and microalgae and changed seawater chemistry [14], [15]. The combined effects from warming and nutrient enrichment is thus of great interest in shallow-water sediment systems and is the main topic of this study.

Coastal eutrophication has gained much attention during the last decades. In shallow bays, increased nutrient loading can ultimately result in a shift in dominance from benthic to pelagic primary production [13], [16], [17]. In most areas of the ocean, the extent and duration of phytoplankton production is constrained by the availability of nitrogen e.g. Antia et al. [18]. The overall fate of nitrogen and biogeochemical pathways during N-transformations have therefore received significant attention during the last decades [17]. However, the effects of nutrient enrichment on nitrogen cycling in shallow-coastal areas are still not completely understood, let alone when combined with globally increasing temperature [13]. In shallow-water coastal environments, net concentrations and nitrogen speciation in pore water and overlying bottom water are regulated by a delicate balance between numerous processes mediated by sediment-living microorganisms, such as mineralization, nitrification, denitrification, anaerobic ammonium oxidation and dissimilatory nitrate/nitrite reduction to ammonium [19], [20]. It has been predicted that an increased supply of nitrogen, resulting in enhanced production of organic material, would result in a reduced removal of nitrogen from these environments, in essence caused by a reduced potential for denitrification in surface sediment [13], [21].

Coastal eutrophication can be expected to change the metabolic balance of the sediment, ultimately turning shallow-water ecosystems more heterotrophic [13]. Warming could be expected to further strengthen this effect since temperature is positively correlated to the kinetics of biological and chemical processes consuming oxygen. Warming may, however, affect a range of autotrophic and heterotrophic processes such as respiration and mineralization [22], [23]. The trophic state of the sediment has been found to be a suitable proxy for the nutrient balance and net fluxes of nutrients between sediment and overlying bottom water [24]–[26].

It has been frequently suggested that warming will exacerbate the effects of local stressors, such as nutrient loading, in the aquatic environment [27], [28]. Hitherto, most studies on the combined effects of warming and nutrients originate from pelagic systems or from benthic vegetated areas with macroalgae or seagrasses [29]–[31]. Among the few published studies on “bare” sediments, none was able to demonstrate interactive effects of warming and nutrient enrichment [32], [10], although single effects by warming and nutrient enrichment were observed. In the study by Boros et al. [32] nutrient addition increased sediment redox potential indirectly by an increase in phytoplankton biomass that efficiently removed macrophytes through shading. Further, Fitch et al. [10] demonstrated increased sediment-water ammonium fluxes and community respiration during warming and addition of organic matter. Neither of these studies, however, considered the activities of benthic microalgae. Since benthic microalgae drive autotrophic processes, and also control important heterotrophic processes in the sediment, their activity and biogeochemical implications need to be included when studying the combined effects of warming and nutrients. Like nutrient enrichment, warming has been shown to affect the composition of phytoplankton [33], [34]. Microcosm experiments with natural inoculum of benthic microalgae, involving raised temperature and nutrient supply ratios, suggested that warming accelerated competitive exclusion, changing species dominance [35]. A change in the composition of the benthic microalgal community due to warming and nutrient enrichment was therefore hypothesized in the present study.

Here, we include processes driven by both autotrophic and heterotrophic components of the microbial community. Our aim was to study whether a predicted temperature rise of 4°C above ambient (predicted by IPCC according to the A1F1 scenario for the year 2100 [1]), in combination with moderate nutrient addition, simulating a realistic inorganic nutrient load in a subtidal shallow-water sediment systems [36], would change the structure and function of a shallow-water sediment system. For this we used intact sediment cores placed in a semi-outdoor flow-through mesocosm facility, making it possible to implement both a controlled warming treatment and mimic the natural temperature fluctuations of the adjacent environment. More specifically, we hypothesized that an increased water temperature in combination with a moderate nutrient enrichment would result in (1) a shift in the trophic state from autotrophy to heterotrophy and a generally strengthened net heterotrophy, (2) changed rates and pathways of nitrogen cycling, and consequently a change in the filter function of the sediment, (3) an altered benthic algal community, and (4) interactive, mainly non–additive effects from warming and nutrient enrichment.

## Materials and Methods

### Ethics Statement

No permits or approvals were required for the sampling since the study area is not protected or private. Further, no protected species were sampled during the sediment collection.

### Approach and Experimental Overview

The experiment started in October 2009 and was terminated in November 2009 (5 weeks duration). Forty-eight intact sediment cores (i.d. 25 cm) were incubated in a flow-through system in a greenhouse and exposed to either seawater at ambient temperature or seawater heated 4°C above the ambient. Half the number of cores was also exposed to nutrient additions alone or in combination with heated seawater. Both functional (ammonium production rates and denitrification in the sediment, as well as benthic fluxes of oxygen and inorganic nutrients) and structural variables (biomass of benthic microalgae and sediment characteristics) were measured on 4 sampling occasions, with no repeated sampling. Sediment–water fluxes were measured both during day and night. After flux measurements, sediment variables were sampled from the top 0.5 cm of the sediment surface.

### Sediment Sampling and Experimental Set-up

Sediment for the experiment was collected with an Olausson box corer (30 cm × 30 cm) at a depth of ∼2 m in Munkeby Bay (58°14′N, 11°32′E) on the west coast of Sweden. Cylinders of black Acrylonitrile Butadiene Styrene plastics (height = 25 cm, i.d. = 25 cm) were used to sample one core from each box-core sample and were later also used for sediment-water incubations. The flow-through set-up, with surface water supplied directly from the Gullmar Fjord, was placed in a greenhouse to allow close to natural light and temperature conditions, as well as protection from precipitation and birds. Seawater was continuously pumped into two elevated 1000-L isolated dark and covered plastic containers placed just outside the greenhouse (turnover time 0.7 h^−1^). From these tanks water was supplied with a flow rate of ∼20 L h^−1^ to each experimental cylinder after passing a 1-mm mesh mounted in cartridge-filter holders (turnover time 4 h). Incoming seawater was monitored for salinity, temperature, pH, oxygen and water flow in the two elevated tanks and in each experimental cylinder. The depth of the overlying water in the cylinders was ∼10 cm, corresponding to a water volume of ∼5 L per cylinder. Four out of 52 sampled sediment cores (cylinders) were used for initial sampling, and the rest (48 cylinders) were allocated to four treatments: (1) ambient temperature, (2) warming only, (3) nutrient enrichment only, and (4) both warming and nutrient enrichment. In addition to the initial sampling (day 1), incubation and sampling were done on days 8, 28 and 38, with 4 replicates per treatment. Manipulations of seawater temperature were performed by using an immersion-heater (6 kW, Energi Ekonom i Höör) mounted in one of the two elevated tanks and connected to a computerized control unit as described by Alsterberg et al. [37]). Sensors in two experimental cylinders (ambient and heated) measured temperature continuously, making it possible to keep incoming seawater 4°C above the temperature of the ambient seawater. A high flow and turnover of incoming water kept the temperature within each experimental cylinder stable and hence unaffected by changes in outside air temperature. Nutrients were added continuously, applying an in-situ enrichment technique using fertilizer pellets [38]. The pellets were placed in mesh bags at the water inlet of each cylinder, where they released inorganic nutrients to the surrounding water. To initiate the nutrient treatment, non-coated pellets were used (Hydro Agri OptiCrop NPK 21-3-10, 5 g). These pellets dissolved within a few days and were replaced by slowly dissolving coated pellets (Plantacote 4 M mix, 15 g). The slowly dissolving pellets where at the end of the experiment almost completely dissolved (∼90%), but since the leakage of nutrients depends on diffusion gradients, changes in pellets surface area should not have affected the nutrient leakage during the time of the experiment. The total supply of nutrients in each cylinder corresponded to ∼80 mmol N m^−2^ day^−1^ and ∼20 mmol P m^−2^ day^−1^. The resulting nutrient concentrations were similar to those used in previous experiments simulating nutrient enrichment [37], [39], [40].

### Structural Variables

The water content of the sediment was calculated from the weight loss after drying ca. 5 mL of wet sediment for 24 hours at 60°C. Sediment content of chlorophyll *a* (Chl *a*) was used to estimate the biomass of benthic microalgae. For Chl *a* content, duplicate sediment samples (5 mm deep) were taken from each replicate cylinder, using a 5 mL cut-off plastic syringe (area 0.785 cm^2^). The sediment was immediately frozen (−20°C) until analysis and further handled as described in Alsterberg et al. [37], applying the method by Lorenzen [41], which corrects for degradation products of Chl *a* (pheopigments). For microalgal counts, two samples from the top 5 mm sediment were taken from each replicate cylinder with a cut-off 2-mL syringe (area 0.594 cm^2^) and stored frozen (−20°C). After thawing, the samples were handled and cells counted using epifluorescense microscopy. Cells were identified to genus level and allocated to size groups. Cell numbers were counted only for day 28 because results from previous experiments have suggested that microalgal composition in natural sediments responds rather slowly – and weakly – to moderate treatments [42], [43]. Moreover, benthic diatoms grow well in a large range of temperature [44] and there was either no significant effects on the microalgal composition in a previous warming experiment using sediment from the same study site [38].

### Functional Variables

Benthic fluxes of oxygen and inorganic nutrients (ammonium, nitrate+nitrite – referred to as nitrate), dissolved inorganic phosphorus (DIP), and silicate) were measured in 16 randomly chosen cylinders (4 per treatment) on days 8, 28 and 38. During sampling, the water flow was turned off and the experimental cylinders were sealed with transparent plastic lids. The overlying water during this ‘batch’ incubation was mixed by a Teflon-coated stirring bar (∼60 rpm) attached to the lid. Benthic fluxes were determined from concentration changes in the overlying water from the start and stop of incubations. Daytime incubations were made between ∼11∶00 h and ∼13∶00 h and night incubations between ∼22∶00 h and ∼04∶00 h. Oxygen concentrations were determined using the Winkler technique and nutrient concentrations were determined by an autoanalyzer (Smart Chem, Westco Scientific Instruments) according to standard colorimetric procedures [45]. Hourly benthic fluxes were converted to daily (24-h) rates by multiplying hourly light fluxes by the number of daylight hours, and hourly night fluxes by the number of dark hours, and then adding these two numbers (light data from Sven Lovén Center of Marine Sciences – Kristineberg, Sweden).

Oxygen fluxes during day and night were used as measures of net primary production (NPP) and community respiration (CR). Gross primary production (GPP) was calculated by adding community respiration (CR) in the dark to NPP. Daily values of NPP were calculated by multiplying oxygen flux rates by a light factor calculated as the ratio between the daily irradiance and the irradiance during the incubation period. The 24-h net oxygen flux was calculated by adding NPP for the light period and CR during the dark hours. For practical reasons, CR in the light was assumed to equal CR in the dark. Oxygen production was also used to calculate the theoretical nitrogen assimilation (N demand) by benthic microalgae, using 80% of GPP, a photosynthetic quotient (PQ) of 1.2 [46] and a C/N ratio of 9, for justification of a ratio above Redfield, see [25], [47].

For potential denitrification, 2 ml of sieved sediment was added to 10 ml gas-tight exetainers flushed with helium and pre-incubated for 24 hours at in-situ temperature to remove oxygen and nitrate present in the sediment. Eighty µl stock solution of ^15^NO_3_ (Na^15^NO_3_, 99.6 atom %, Europa Scientific Ltd) was added to each exetainer, resulting in a concentration of ∼50 µM in the sediment pore water. Samples were shaken and incubated in the dark for 2.5 hours. The incubation was terminated using ZnCl_2_. Sediment samples were analyzed at the National Environmental Research Institute, Denmark, and potential denitrification was calculated according to Thamdrup and Dalsgaard [48].

### Nitrogen Mineralization

Nitrogen mineralization was assessed in two ways. (1) Rates of nitrogen mineralization (ammonification) were estimated from the net mobilization of ammonium to the pore water during closed sediment incubations, i.e. the net change in concentration of ammonium with time (N_mineralized_ = NH_4_^+^_mobilized_) corrected for reversible adsorption with sediment particles [49]. This will in the following text be referred to as ‘nitrogen mineralization’. Fifty mL centrifuge tubes were, after pretreatment with N_2_, filled with sieved (1 mm mesh) surface (0–2 cm) sediment and incubated under anoxic conditions in the dark at ambient (range 8–12°C) and at raised temperature (range 12–16°C). Anoxic and dark conditions were ensured by incubating the tubes (n = 3 for each treatment) in a large (50×80×40) bucket filled with sediment from the sampling site. The incubation was terminated after ∼24 hours by removing the tubes from the incubation bucket and separating pore water from the sediment by centrifugation. The pore water was filtered (0.45 µm) on-line and concentrations of ammonium determined using an autoanalyzer (Smart Chem, Westco Scientific Instruments) according to standard colorimetric procedures [45]. (2) Rates of N-mineralization were calculated from measured sediment–water nitrogen fluxes and denitrification (N_mineralized_ = NH_4_^+^_flux_+ΣNO_3_^−^+NO_2_^−^_flux_+denitrification). These rates will be referred to as ‘flux-based nitrogen mineralization’.

### Statistical Analysis

Data were analyzed using a mixed model ANOVA, where temperature (ambient and heated) and nutrient status (no nutrients added, nutrients added) were included as fixed factors, while the factor ‘time’ (3 sampling occasions) was included as a random factor. The choice of ‘time’ as a random factor was justified by the fact that we wanted to view the general temporal variation, without focusing on specific sampling days. Data from initial cores were not included in the statistical analysis since they only served as a starting point of the experiment. The possibility of making a type I error was set to α 0.05. All data were checked for normality and homogeneity of variances using box, residual and Q-Q plots. Data were normally distributed and although homogeneity of variance was not always met, data were not transformed. Statistical analyses were performed in the R environment [50]. Three-way permutational multivariate analysis of variance (PERMANOVA) with temperature and nutrients as fixed factors was used to analyze the composition (relative abundance based on cell counts) of benthic microalgal community. The raw data was not transformed but standardized through normalization. The analyses were run using the program PERMANOVA version 1.6 [51].

## Results

**Figure 1:**
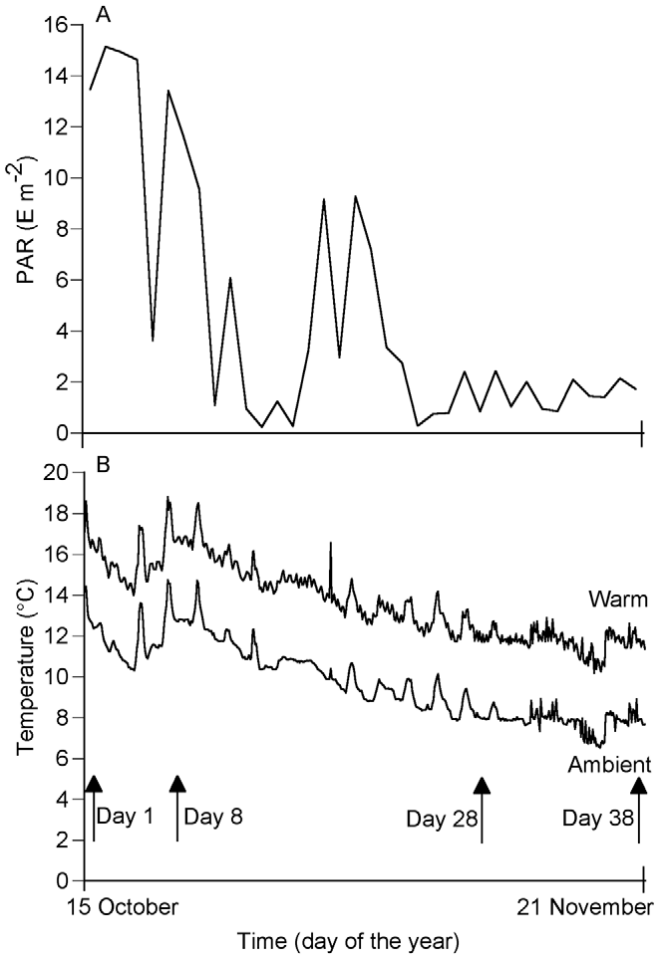
Total insolation during the time of experiment (A) and the temperature of ambient and heated seawater measured every hour during the experimental period (38d) (B). Arrows indicate sampling days.

Ambient daily irradiance was 13–16 E m^−2^ in the beginning and ∼2 E m^−2^ at the end of the experiment (Figure 1A). Ambient seawater temperature decreased from 14.2°C to 7.7°C and from 17.1°C to 11.4°C in the heated treatment (Figure 1B). The mean temperature difference between the two treatments was 3.9°C. The nutrient enrichment significantly increased the concentrations of DIN in the overlying water by 50% and DIP by 30% (Table 1).

**Table 1:** Concentrations of dissolved inorganic nitrogen (DIN) (ammonia and nitrate+nitrite) and dissolved inorganic phosphate (DIP) in the overlying water for each treatment and sampling day, measured at the start of the incubations.

		Experimental treatments			
		Ambient	Warming	Nutrient	Warming × Nutrient
Day 1	DIN	1.5±0.2			
	DIP	0.3±0.2			
Day 8	DIN	2.3±0.6	3.6±3.0	5.2±0.6	6.6±2.5
	DIP	0.6±0.04	0.7±0.2	0.9±0.2	1.2±0.8
Day 28	DIN	6.0±3.4	3.5±0.4	7.0±0.9	5.0±1.1
	DIP	0.8±0.06	0.7±0.07	0.9±0.03	1.2±0.5
Day 38	DIN	8.1±1.9	9.2±0.6	11.6±1.4	11.3±1.6
	DIP	0.5±0.04	0.5±0.04	0.7±0.02	0.6±0.03

Shown are mean concentrations ± SD in *µ*mol L^−1^ (n = 4).

### Benthic Primary Production and Community Respiration

**Figure 2:**
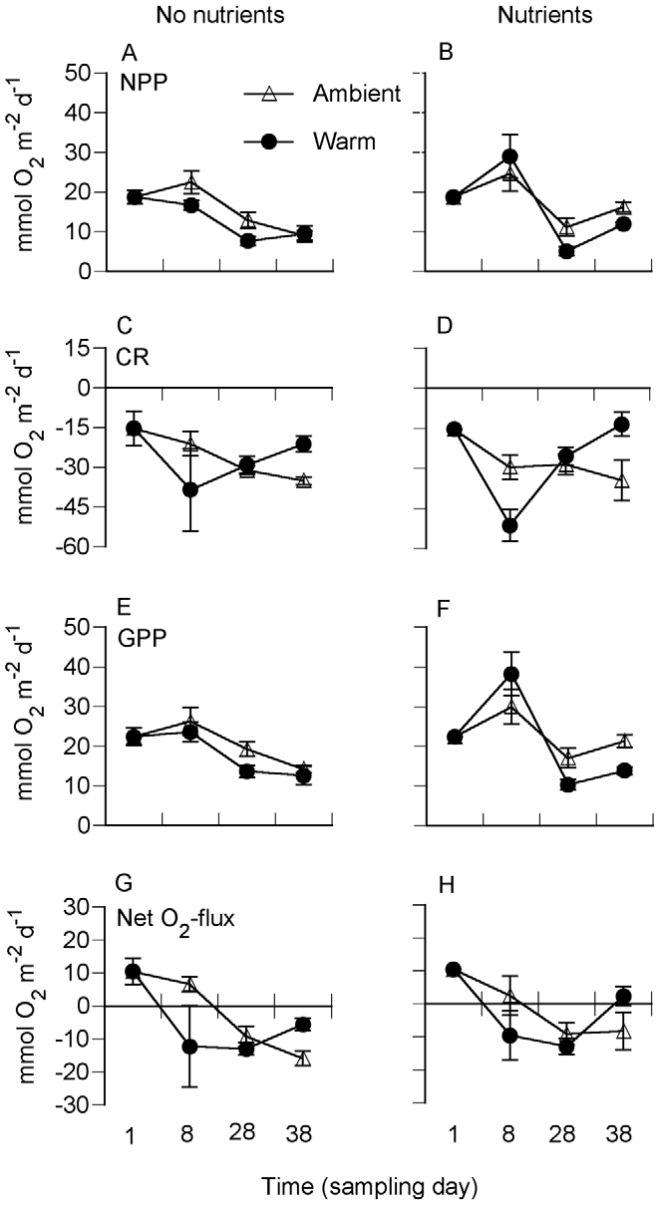
Daily sediment–water oxygen fluxes: Net primary production (NPP) (A, B), community respiration (CR) (C, D), gross primary production (GPP) (E, F) and 24-h net oxygen fluxes (G, H) at ambient nutrient level (left panels) and with nutrients added (right panels), at ambient seawater temperature and in heated seawater. Shown are means ± SE (n = 4).

Primary production (NPP and GPP) was significantly stimulated by the nutrient addition (Nu × D), but not by warming, with the exception of GPP being almost significantly affected by warming (W × D, *p* = 0.059) (Figure 2A, 2B, 2E, 2F, Table 2). Warming, on the other hand, initially stimulated community respiration, but reduced respiration towards the end of the experiment (Figure 2C, 2D). There was no significant interaction between warming and nutrient addition (Table 2). The sediment system was initially net autotrophic (oxygen producing) but warming shifted the trophic balance to net heterotrophic (oxygen consuming; Figure 2G, 2H). Later, however, the response pattern differed between the two temperature treatments. While the sediment exposed to ambient temperature continued towards a more heterotrophic state, the degree of heterotrophy decreased in the heated treatment. There was even a tendency that the exposure to both warming and nutrient enrichment shifted the system to autotrophy towards the end of experiment (Figure 2G, 2H, Table 2).

**Table 2:** Effects of warming and nutrient enrichment tested by mixed-model ANOVA with the fixed factors temperature (W) and nutrient enrichment (Nu) and the random factor day (D).

**Warming × Nutrient enrichment**	***p***	0.082	0.771	0.130	0.816	0.406	0.088	0.504	0.322	**0.018**	0.793	**0.009**	0.130	0.431	**0.017**
	***F***	10.6	0.1	6.2	0.07	1.1	9.7	0.6	1.7	54.5	0.09	102.3	6.2	0.9	4.5
	**MS**	169104.5	6.6	93.1	5.2	16.4	1131433	32764.1	305414.9	63111.6	33.4	1.2×10^6^	93.1	8612.9	2.4
	**df**	1	1	1	1	1	1	1	1	1	1	1	1	1	2
	**Factor**	W × Nu	W × Nu	W × Nu	W × Nu	W × Nu	W × Nu	W × Nu	W × Nu	W × Nu	W × Nu	W × Nu	W × Nu	W × Nu	W × Nu × D
**Nutrient enrichment**	***p***	**0.032**	**0.035**	0.891	**0.014**	0.467	**0.026**	0.866	**0.035**	0.761	0.061	**0.028**	0.891	0.837	**0.042**
	***F***	3.8	3.7	0.02	4.8	0.7	4.1	0.4	26.7	1.9×10^−4^	14.9	3.9	0.02	0.05	3.5
	**MS**	792754	95.4	46.9	144.5	69.5	3×10^6^	260677	119926	5.1	368.1	3.9×10^6^	46.9	14.9	1.8
	**df**	2	2	1	2	1	2	1	1	1	1	2	1	1	2
	**Factor**	Nu × D	Nu × D	Nu	Nu × D	Nu	Nu × D	Nu	Nu	Nu	Nu	Nu × D	Nu	Nu	Nu × D
**Warming**	***p***	**0.027**	0.203	**<0.001**	0.059	**0.003**	**0.048**	0.082	0.730	0.761	**0.029**	0.154	**<0.001**	**0.005**	**0.021**
	***F***	3.9	3.5	26.6	3.1	6.9	19.1	2.7	0.1	0.1	3.9	5.03	26.6	6.1	4.4
	**MS**	834374	10357.1	1960.7	91.2	673.7	6×10^6^	736366	30952.2	412.1	1001.8	6×10^6^	1960.7	31240.9	2.3
	**df**	2	1	2	2	2	1	2	1	1	2	1	2	2	2
	**Factor**	W × D	W	W × D	W × D	W × D	W	W × D	W	W	W × D	W	W × D	W × D	W × D
	**Variables**	Sediment Chl *a*	NPP	CR	GPP	Net O_2_ flux	NH_4_^+^ flux	NO_3_^−^+NO_2_^−^ flux	Si flux	DIP flux	Mineralization_inc_	Mineralization_flux_	N assimilation	Pore water NH_4_^+^	Denitrification

df, degrees of freedom; MS, mean square; *F*, *F*-ratio; *p*, *p*-value. Mineralization_inc_ is mobilization of ammonium measured in closed incubations, mineralization_flux_ is the sum of fluxes of ammonia, nitrate+nitrite and nitrogenous gas. P-values for significant effects of experimental treatments are shown in bold.

### Cycling of Inorganic Nutrients

**Figure 3:**
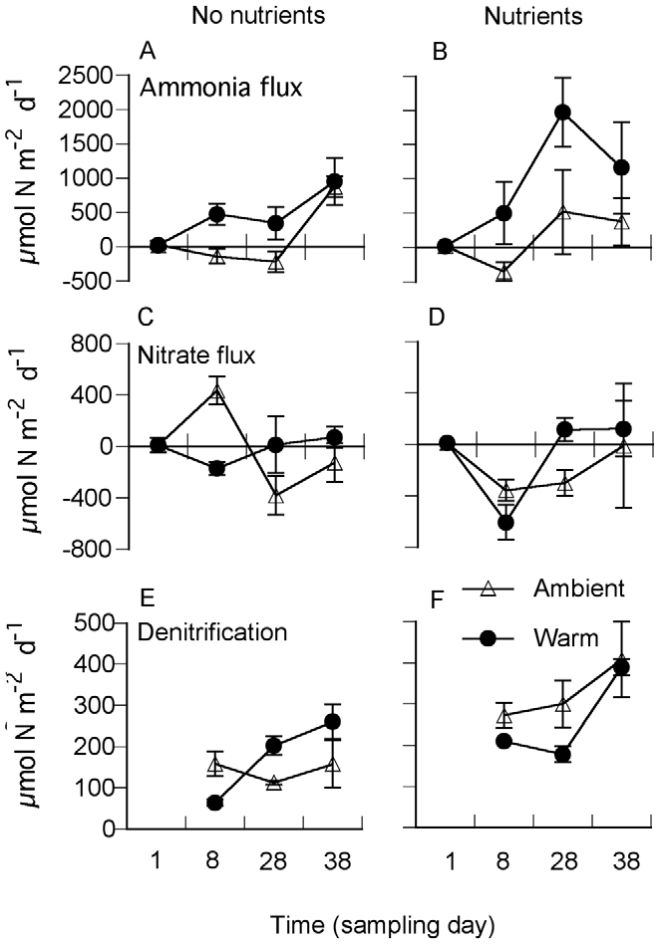
Daily sediment–water nitrogen fluxes: Ammonium (A, B), nitrate+nitrite (C, D) and denitrification (E, F) at ambient nutrient level (left panels) and with nutrients added (right panels) at ambient seawater temperature and in heated seawater. Shown are means ± SE (n = 4). Note different scales on y-axes.

**Figure 4:**
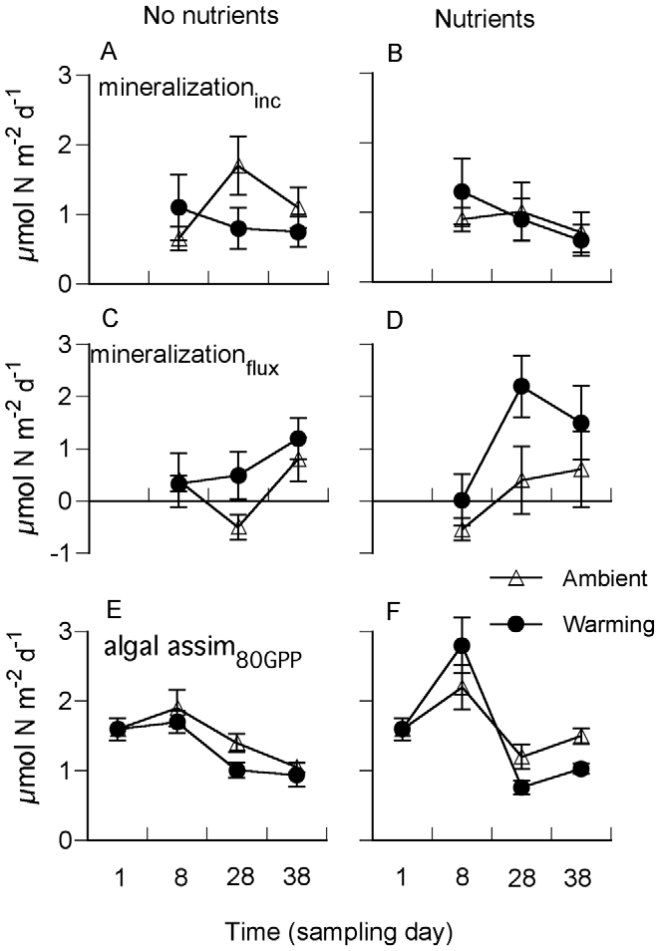
Nitrogen mineralization measured as NH_4_^+^ mobilization in closed sediment incubations (mineralization_inc_) (A, B), net nitrogen mineralization calculated from fluxes of ammonium, nitrate+nitrite and denitrification (mineralization_flux_) (C, D) and algal assimilation based on 80% of GPP (algal assim_80GPP_) (E, F) at ambient nutrient levels (left panels) and with nutrients added (right panels), at ambient seawater temperature and in heated seawater. Shown are means ± SE (n = 4).

All measured processes during nitrogen cycling were affected by the experimental treatments, but in different ways (Table 2). A significant warming – nutrient interaction (W × Nu) was found for denitrification (Figure 3E, 3F) and flux-based nitrogen mineralization (Figure 4C, 4D). The combined effect of temperature and nutrients on denitrification was synergistic, increasing rates by a factor of ∼3. Also flux-based mineralization increased synergistically when temperature and nutrients were combined (W × Nu) (Figure 4C, 4D, Table 2). Rates of ammonium mobilization to the pore water were depending on warming and sampling occasion (W × D) (Figure 4A, 4B, Table 2). Calculations of benthic microalgal assimilation of nitrogen (theoretical N- demand based on 80% GPP) was significantly affected by nutrient addition depending on the day of sampling (D×Nu) (Figure 4E, 4F, Table 2).

Sediment–water fluxes of ammonium were affected by both warming and nutrient additions, but without interaction. Nitrate fluxes were not affected by either temperature or nutrients (Figure 3, Table 2). Warming alone increased the efflux of ammonium from the sediment to the overlying water during the entire experiment and so did nutrient addition. Warming thus turned the sediment from a sink to a source of ammonium.

**Figure 5:**
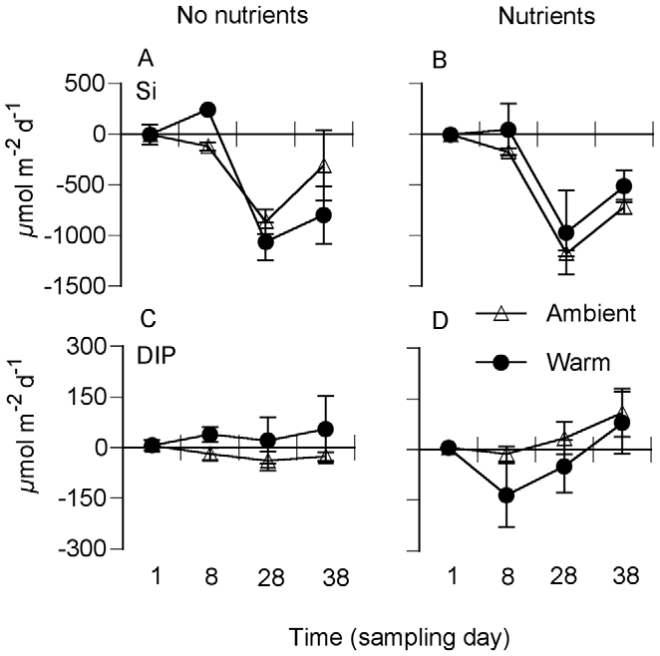
Daily sediment–water fluxes for silica (A, B) and dissolved inorganic phosphorus (DIP) (C, D) at ambient nutrient level (left panels) and with nutrients added (right panels) at ambient seawater temperature and in heated seawater. Shown are means ± SE (n = 4).

In addition to denitrification and flux-based N mineralization, DIP flux was the only other variable for which a (non-additive) warming–nutrient interaction (W × Nu) was found. This effect was, however, antagonistic. So when nutrient addition was combined with warming it turned the sediment from a source to a sink for DIP (Figure 5C, 5D, Table 2). Nutrient addition alone increased the uptake of silicate, while temperature had no effect on silicate fluxes (Figure 5A, 5B, Table 2).

### Benthic Microalgae

**Figure 6:**
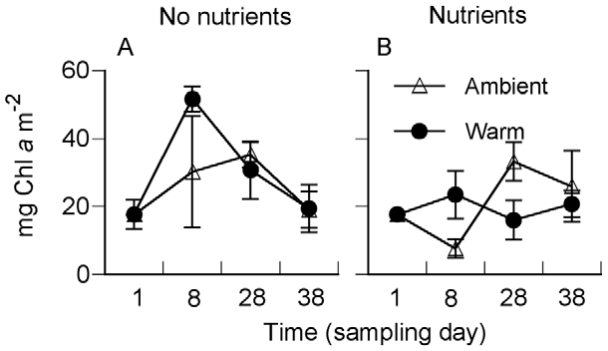
Chlorophyll *a* concentration in the top 5 mm of sediment at ambient nutrient level (A) and with nutrients added (B), at ambient seawater temperature and in heated seawater. Shown are means ± SE (n = 4).

Warming initially increased the Chl *a* content of the surface sediment (Figure 6A, Table 2). The effect of nutrient enrichment on Chl *a* was less consistent (Figure 6B, Table 2), and at the end of the experiment no significant effects remained. The benthic microalgal community consisted of diatoms and cyanobacteria, with diatoms dominating cell counts. In contrast to our previous warming experiments in spring [38], no floating microalgal mat was formed in the experimental cylinders. The multivariate analysis based on the relative abundance of 64 microalgal taxa/size groups on day 28 did not reveal a significant change in the composition. Therefore, and due to the time-consuming procedure, no further samples were counted. Occasionally, the cyanobacterial genus *Oscillatoria* contributed up to ∼90% of the photosynthetic biomass because of its large thrichome volume. There was a tendency of a higher cyanobacterial biomass in the warm treatment than in the ambient and nutrient-only treatments, respectively. However, these differences were not statistically significant. No cyanobacteria were observed in the warming–nutrient treatment.

## Discussion

Previous experiments have shown that the net response of natural, multi-trophic sediment systems to various natural and anthropogenic stressors often change over time due to the simultaneous operation of both direct and indirect effects [38], [52], [53]. Despite the fact that time-dependent effects were observed also in our experiment, the following general results were distinguished: (1) Although a shift from net autotrophy to net heterotrophy occurred earlier with warming (Figure 2G), heterotrophy was not generally strengthened when compared with the exposure to ambient temperature. In fact, warming pushed the system back towards autotrophy at the end of the experiment (Figure 2G, 2H). (2) Rates and pathways of nitrogen cycling were affected by both warming and nutrient enrichment, thus supporting our second hypothesis. (3) Although the biomass of benthic microalgae was affected by the treatments, our hypothesis about a changed composition of the algal community was not supported. (4) Finally, some non-additive interaction effects were observed, partially supporting the expectation of synergism between warming and nutrient addition, though only for two variables, both related to nitrogen cycling (nitrogen mineralization and denitrification).

As our experiment was performed in the autumn, the sediment system was naturally exposed to gradually cooling seawater and a decreasing light level for benthic photosynthesis (Figure 1A, 1B). This experimental period was chosen on purpose to contrast the opposite situation in a previous spring experiment, with naturally increasing temperature and light level, and initially autotrophic sediment [38]. In autumn, the silty shallow-water sediments in our study area were expected to be heterotrophic due to enhanced mineralization and respiration rates [54], [55], resulting in higher net mobilization of nutrients to the pore water and a flux of nutrients from the sediment to the overlying bottom water [55].

### Warming and Benthic Trophic State

The hypothesis that warming would induce a shift in the trophic state was based on the theoretical framework where heterotrophic processes have been observed to respond more strongly and rapidly to warming than autotrophic processes [22], [56]. After one week, the sediment had turned net heterotrophic in the heated treatments because of increased community respiration, while autotrophy was retained in the experimental containers exposed to ambient temperature (Figure 2G). Primary production, on the other hand, was stimulated by nutrient addition but not by warming. This result was also corroborated by the increased flux of silica from the overlying water to the sediment, which likely reflected enhanced activity of benthic diatoms [57]. The 24-hour net oxygen flux (Figure 2G, 2H) suggested that, while the degree of heterotrophy progressively increased at ambient temperature, heterotrophy later decreased at elevated temperature. At the end of the experimental period, and as a consequence from warming, the 24-hour net oxygen flux even indicated autotrophic sediments. The explanation for this opposite trend in the trophic state with warming could be that an initial short-term increase in community respiration and mineralization resulted in a shortage of labile and easily degradable organic material with time of incubation and this shortage resulted in progressively decreasing bacterial activity. Such a scenario would imply a short-term significant change towards a heterotrophic state with increased mineralization rate followed by reduced net mineralization rate in the sediment, and ultimately perhaps autotrophic sediments. Thus, unexpectedly, warming in fact may have counteracted the hypothesized shift in trophic state.

### Rates and Pathways of Nitrogen Cycling

Warming and nutrient enrichment were hypothesized to change rates and pathways of nitrogen cycling. Nitrogen mineralization, benthic fluxes of ammonium and rates of potential denitrification increased during warming (Figures 3 and 4). The progressively increasing accumulation of ammonium in the pore water during the closed sediment incubations also reflected a stimulated nitrogen mineralization with warming and nutrient addition. Combining warming and nutrient enrichment further increased nitrogen mineralization (fluxed based) and denitrification, resulting in a synergistic effect. Rates of nitrogen mineralization have been shown to correlate positively with temperature and labile organic matter [58]. Despite ongoing warming throughout the experimental period, the rate of mineralization was reduced with time during the closed sediment incubations (Figure 4A, 4B). Quite in parallel to the earlier discussion on warming effects on the net oxygen balance (trophic state), a progressively decreasing pool of reactive organic matter would, in the long term (i.e. late autumn–winter), result in reduced rates of nitrogen mineralization.

Potential denitrification was stimulated synergistically by the combination of warming and nutrient enrichment. Denitrifying bacteria have a rather wide temperature range (0–35°C) [59], [60], and within ecologically relevant concentrations, denitrification follows first order kinetics with respect to nitrate concentrations [61]. The observations that nitrogen (i.e. NO_3_^−^) addition stimulated denitrification indicated an increased transport of nitrate from the overlying water to the zone of denitrification in the surface sediment. Also, denitrification rates were inversely related to nitrogen mineralization (Figures 3 and 4). This observation can be interpreted as reduced nitrification–coupled denitrification, as oxygen penetration and the sediment volume available for aerobic ammonium oxidation (nitrification) are reduced with increasing rates of organic matter mineralization. We did not measure nitrification rates directly, but budget balancing fluxes of nitrate and rates of denitrification suggested no change in rates of nitrification due to warming and nutrient enrichment (Figure 3). A synergistic response of the denitrifying bacteria was therefore related to an increased temperature in combination with a preferential uptake of nitrate added to the overlying water, and not to increased nitrification fuelling nitrification-coupled denitrification.

One major function of shallow-water areas is their inherent capacity to retain nitrogen and hence function as a filter between land and sea [13]. Nitrogen mineralization has been found to respond faster to temperature than processes that incorporate ammonium, such as assimilation by benthic microorganisms [62], [63]. This scenario also seemed to apply to our system. The fact that mineralization, and subsequent efflux of ammonium, increased more than processes contributing to ammonium assimilation and retention of nitrogen (nitrification, denitrification and algal assimilation), suggested that there was a preferential response of the microbial community driving nitrogen mineralization. Thus, the potential retention capacity of the sediment system in the coastal nutrient filter could change, so that the source function would be strengthened. However, depth-integrated rates of ammonium production during the closed sediment incubations exceeded the measured benthic efflux of ammonium by a factor of 1.5–4 (Figure 3A, 3B, 4C, 4F). Although part of this discrepancy can be explained by methodology and that an important fraction of nitrogen mineralization takes place below 2 cm sediment depth, assimilation by microorganism in the surface sediment is most probably a major sink term.

Nitrogen assimilation by benthic microalgae can be expected to be a large – often the major– temporal sink of nitrogen in shallow-water sediments [25], [64], [65]. Estimated mean algal assimilation rates of 1.2–1.6 mmol N m^−2^ day^−1^ were within the same order of magnitude as the mean nitrogen mineralization measured in the closed sediment incubations (1.2–1.4 mmol N m^−2^ day^−1^) (Figure 4). Algal assimilation rates exceeded mean rates of denitrification by a factor of 5–12 (Figure 3E, 3F), which also suggested incorporation of nitrogen into biomass rather than nitrogen removal from the sediment system by denitrification. So, even though significantly changed rates and pathways of nitrogen were observed, the quantitative net effect on the benthic N flux was not very strong. The reasons could be that the increased mineralization rate did not last long and that the benthic microalgae at the sediment surface efficiently intercepted the flux of ammonium out of the sediment. Without the presence of benthic microalgae the efflux would have been several magnitudes higher. Therefore, despite an increased temperature and nutrient availability, benthic microalgae seemed to sustain the filter function of the investigated illuminated sediment systems.

### Weak Response of the Benthic Microalgal Community

In phytoplankton communities, warming has been found to influence the species composition by shifting the community from microplankton to nanoplankton [66]. Similarly, the rapid response of phytoplankton growth to nutrient enrichment is typically accompanied by an altered community composition [67]. Such alterations did not, however, apply to the microphytobenthic community in our experiment. Although some time-dependent effects of warming and nutrient enrichment on sediment Chl *a* were observed, the response of the benthic microalgae was limited. The tendency towards a higher cyanobacterial biomass with warming alone could be a sign of cyanobacteria being favored by higher temperature, but not by nutrients.

As discussed previously [38], the reason for the lack of evident compositional changes might have been that the well-developed benthic microalgal community was already very dense and fully developed. As a consequence, the growth of microalgal species potentially favored by warming or nutrients would not result in detectable compositional changes. One additional feature that distinguishes benthic microalgae from phytoplankton is the lack of major – often predictable – seasonal changes in the composition. Although seasonal differences exist also in benthic algal communities [68], the same taxa can be found throughout the year, along with a high compositional diversity [69], [70]. Hence, a moderate warming cannot be expected to quickly cause a substantial change in the composition of benthic microalgae. This conclusion is also supported by findings that the growth rate of 25 common benthic diatoms was not affected by temperatures between 10 and 30°C [45]. However, indirect, food-web-mediated effects on the composition could be expected, provided that grazers are affected by the treatments [71]. This emphasizes the importance of using intact benthic systems in experiments and highlights that prediction of combined warming – nutrient effects on microbenthic communities cannot be based on results from phytoplankton studies only.

### Additive and Non-additive Interactive Effects

It was hypothesized that the combination of warming and nutrient enrichment would result in mainly non-additive interactive effects on the shallow-water system. However, with the exception of non-additive effects on benthic nitrogen mineralization and denitrification (synergistic), and fluxes of DIP (antagonistic), we found mainly individual effects of warming and nutrient enrichment. The scarcity of interactive effects could be related to the type of sediment used. The availability of nutrients in silty sediments is high [12], [24] when compared with for example sandy sediment systems, which are often nutrient limited [72], [73]. Increased mineralization at warming further increased the availability of nutrients in the pore water. Therefore, although significant effects of nutrient enrichment of the overlying water were found for algae-related processes (primary production and silica uptake) (Figures 2, 5A, 5B), these effects were in most cases not strong enough to create synergistic warming–nutrient effects.

Nitrogen mineralization and denitrification were associated with a synergistic mode of interaction. We believe that this result is related to the type of microscopic organisms (heterotrophic and chemoautotrophic bacteria and archeons) driving these processes. As discussed earlier, such microbial communities have been found to respond faster to warming than photoautotrophic organisms [56], [74]. Also previous experiments studying effects of simultaneous multiple stressors on similar sediments, suggested that heterotrophic processes, particularly those controlling nitrogen cycling, were affected more frequently than autotrophic processes, such as primary production [38], [54].

### Tentative Ecological Implications

Our results suggest that warming combined with nutrient enrichment can generate non-additive effects on nutrient cycling, specifically on nitrogen cycling. The mode of interaction is related to the trophic level of the organisms that drive the affected processes. This could imply that rates of heterotrophic processes will increase synergistically in response to the warming–nutrient combination, whereas rates of autotrophic processes are less affected. Moreover, in shallow waters the photoautotrophic component can dampen the ecological consequences of the synergism, contributing to the overall resilience of the sediment system. The response of the sediment system can also depend on sediment type. Sandy sediments are, when compared to silty sediments, more autotrophic and may be less affected than silty sediments. At larger water depths in the photic zone, benthic microalgae become less active due to lower light availability [47], and hence the combined effects of warming and nutrient enrichment can be expected be stronger along with increasing heterotrophy of the sediment. Since temperature increases the rate of mineralization, the transport of nutrients out of the sediment will increase with increasing water depth, providing more nutrients for pelagic production, such as phytoplankton and fast-growing macroalgal mats. Thus, warming may, like eutrophication, contribute to a shift in the type of primary producers, resulting in decreased retention of nutrients in the coastal filter. In the very shallow-water, however, benthic microalgae will continue to sustain the filter-function.
